# Understanding Financial Toxicity in Patients with Head and Neck Cancer: A Systematic Review

**DOI:** 10.1177/11795549221147730

**Published:** 2023-01-23

**Authors:** Mattie Rosi-Schumacher, Shivam Patel, Chandat Phan, Neerav Goyal

**Affiliations:** 1 Department of Otolaryngology—Head and Neck Surgery, Jacobs School of Medicine and Biomedical Sciences, The State University of New York at Buffalo, Buffalo, NY, USA; 2 Pennsylvania State University College of Medicine, Hershey, PA, USA; 3 Department of Otolaryngology—Head and Neck Surgery, Penn State Milton S Hershey Medical Center, Pennsylvania State University, Hershey, PA, USA

**Keywords:** Financial burden, financial toxicity, cost of treatment, cancer survivorship, head and neck cancer, health-related quality of life

## Abstract

**Background:**

Cancer treatment often results in financial burdens for patients including healthcare costs as well as treatment-induced disability leading to “financial toxicity” (FT) and decreased quality of life. The purpose of this review is to describe FT related to head and neck cancer (HNC) treatment, including quantifications of direct and indirect costs and descriptions of measurement tools.

**Methods:**

PubMed, Embase, Cochrane Library, and Web of Science databases were searched to identify articles published before April 2022. Full-text published studies were included if they assessed direct or indirect costs of HNC treatment; studies were excluded if they did not focus on HNC or financial burden. The risk of bias was assessed, and the results of the studies were synthesized.

**Results:**

Database searches yielded 530 unique studies, and 33 studies met the criteria for inclusion. Medical expenses for patients with HNC were higher than for patients with other cancers or controls in several studies. Major surgical procedures, neck dissection, free-flap reconstruction, and intensive care unit admission increased hospital costs. Trimodal therapy with surgery plus chemoradiation represented the most expensive treatment, and chemoradiation increased complication-related health care costs. In several studies, >50% of patients treated for HNC were disabled and did not return to work. One of the greatest contributors to the indirect cost of HNC treatment is the loss of lifetime wages. Patients with HNC are at risk for depression, anxiety, and social isolation, which are linked to a decreased quality of life and treatment non-adherence. The only tools used to assess FT in patients with HNC are the Comprehensive Score for financial Toxicity (COST) and the Financial Index of Toxicity (FIT).

**Conclusion:**

Financial toxicity is highly prevalent among patients with HNC. Further research is needed to validate the assessment tools for quantifying FT in HNC patients.

## Introduction

Biomedical advances in HNC therapy, surgery, and technology have prolonged patient survival, slowed cancer progression, and reduced the use of toxic therapies.^1^ However, treatments can still result in substantial physical, functional, and psychosocial morbidities that decrease the level at which patients can function.^2-5^ In addition, the sizeable out-of-pocket costs for treatments further burden the estimated 15.5 million cancer survivors in the United States.^6,7^

**Figure 1.**
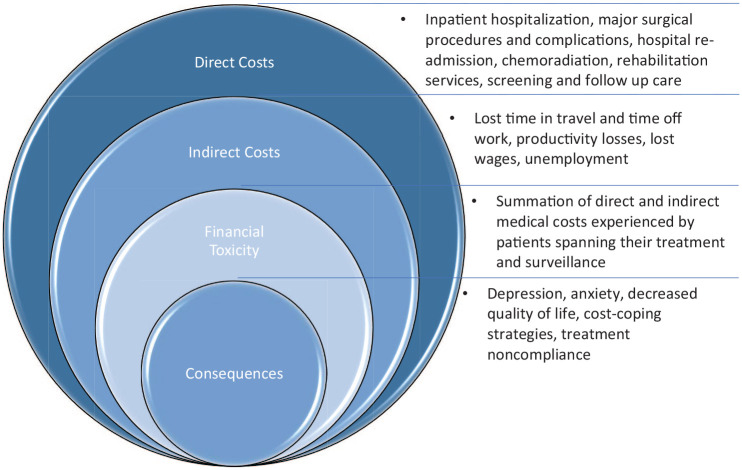
Financial toxicity is multifactorial and encompasses both direct and indirect costs associated with cancer treatment and often leads to adverse consequences for patients’ QOL.

Cancer-related financial burden, or FT, encompasses the direct and indirect medical costs that patients incur throughout their treatment and surveillance (Figure 1). Direct costs are those related to medical costs of cancer treatment, side effect management, and survivorship care, whereas indirect costs are those related to treatment-induced disability, lost productivity, and lost wages. The “toxicity” of these cancer-related financial burdens is reflected in patients’ psychological distress and decreased quality of life (QOL), leading to increased rates of nonadherence to treatment and worse overall disease survival.^8-10^ With approximately 550,000 new cases of HNC worldwide each year^11^ and more than US$3.64 billion in total annual medical costs in the United States,^12^ FT is an important and increasingly prevalent issue. A more thorough understanding of the sources of FT in patients with HNC is needed to design interventions and develop strategies to mitigate this problem.

The aims of this systematic review were to synthesize evidence on the components of financial burden faced by patients with HNC and address the following research questions and objectives: (1) Are patients with HNC more susceptible than patients with other cancers to FT? (2) What are the major factors contributing to the high costs of treatment in HNC? (3) Has FT been shown to adversely affect health-related QOL (HRQOL) in patients with HNC? and (4) To compare the metrics of validated tools that exist to quantitatively assess financial burden specifically in HNC.

## Methods

### Outcomes assessed and eligibility criteria

This study was conducted according to the Preferred Reporting Items for Systematic Reviews and Meta-Analyses (PRISMA) guidelines. We identified articles published from the start of the database until the time of our search in April 2022. Primary outcomes included assessments of direct costs among HNC patients (including inpatient and outpatient services, surgical procedures, hospital costs, treatment modality, and treatment-related complications) and indirect costs (including adverse effects on employment and productivity). Consequences of FT associated with HNC treatment such as the negative impact on QOL and cost-coping strategies are included as a secondary outcome. The last outcome assessed was the use of quantitative assessments specifically addressing FT in patients with HNC. Studies assessing these outcomes during any timeframe of the HNC diagnosis, treatment, and survivorship periods were included in the review. Case reports, commentaries, abstract only, and non-English publications were excluded from full-text review. Studies were excluded if they did not specifically address patients with HNC and financial burden or direct/indirect costs as outlined above.

### Search strategy and study selection

**Figure 2.**
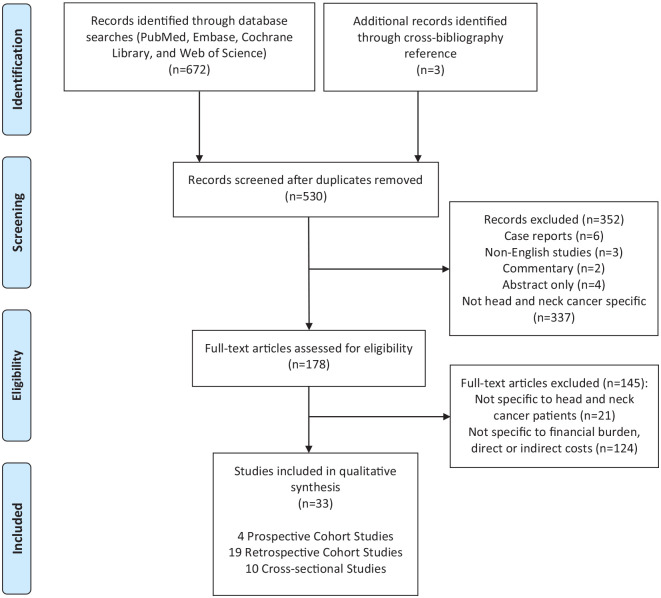
Preferred Reporting Items for Systematic Reviews and Meta-Analyses flow diagram for systematic review methods and article inclusion and exclusion.

Independent searches of PubMed, Embase, Cochrane Library, and Web of Science databases were conducted to identify articles published from the start of the database until the time of our search in April 2022 describing FT in HNC. The keywords used were synonyms for FT (such as financial burden, burden of treatment, financial hardship, financial effects, financial impact, health care costs, cost of treatment, cost of illness, health expenditure, economic burden, and cost of illness) and synonyms for HNC (such as head and neck tumor, neoplasm or carcinoma, cancer of the head and neck, or head and neck surgery) in the title and abstract. These components were used to identify corresponding MeSH terms for search strings in PubMed. Search terms were modified on the basis of keyword availability for each database to ensure a broad scope of study inclusion. See Supplement 1 for a full list of search terms for each database. Additional references were obtained from a manual search of the bibliographies within the retrieved articles. Search results were uploaded to an EndNote database, and duplicates were removed. Subsequently, exclusion and inclusion criteria were applied, and eligible full-text studies were reviewed (Figure 2).

### Data collection

Three independent authors (M.R.-S., S.P., and C.P.) participated in the review process and screened and selected studies for full-text review and inclusion. Discrepancies regarding article inclusion were resolved via discussion among the authors to achieve consensus with input from the senior author (N.G.). For full articles included in the final review, data regarding the study population, study method, tools and outcomes used, figures regarding direct cost of treatment, and objective and subjective indirect cost statistics were collected and tabulated.

### Assessment of bias

Studies were evaluated for risk of bias using the Newcastle-Ottawa Quality Assessment Scale (NOS) by three authors (M.R.-S., S.P., and C.P.) independently, with discrepancies resolved after joint article review and discussion with input from the senior author (N.G.) (Table 1). The NOS includes three categories for quality assessment: selection, comparability, and outcome. Selection was graded on a 4-point scale with 1 point each rewarded if (1) the study cohort was representative of the average HNC population, (2) the selection of the comparison group was from the same community as the exposed cohort, (3) the ascertainment of data was drawn from medical records or structured interview, and (4) the demonstration that the outcome of interest was not present at the start of the study. Comparability was graded on a 2-point scale with 1 point each rewarded if (1) the study provided a control group for non-HNC patients or (2) a group was selected to control for additional relevant factors such as socioeconomic factors, cancer subsite, insurance, or treatment modality. Outcome was graded on a 3-point scale with 1 point each rewarded if (1) the assessment of the study outcome was independent, blinded, or based on objective data from records; (2) follow-up was long enough to assess outcomes; and (3) there was a limited loss of patient data due to attrition.

**Table 1.** Risk of bias assessment using the NOS.

Author	Method	NOS—total (9)	NOS—selection (4)	NOS—comparability (2)	NOS—outcome (3)
Jacobson et al^2^	Retrospective cohort	7	3	2	2
Chen et al^3^	Cross-sectional cohort	5	3	0	2
Massa et al^6^	Retrospective cohort	9	4	2	3
Tribius et al^8^	Prospective cohort	5	2	2	1
de Souza et al^9^	Prospective cohort	6	4	0	2
Lairson et al^13^	Retrospective cohort	6	3	2	1
Chan et al^14^	Retrospective cohort	4	2	0	2
Hennessey et al^15^	Retrospective cohort	6	3	1	2
Adjei Boakye et al^16^	Retrospective cohort	7	4	0	3
Jones et al^17^	Retrospective cohort	3	2	0	1
Tom et al^18^	Retrospective cohort	3	2	0	1
Lang et al^19^	Retrospective cohort	6	3	1	2
Arshad et al^20^	Retrospective cohort	5	3	0	2
Moore et al^21^	Retrospective cohort	6	3	1	2
Brandenburg et al^22^	Retrospective cohort	4	2	0	2
Nonzee et al^23^	Retrospective cohort	6	3	1	2
Kochhar et al^24^	Retrospective cohort	7	3	1	3
Pike et al^25^	Retrospective cohort	8	4	2	2
Semenov et al^26^	Retrospective cohort	6	3	0	3
Beeler et al^27^	Prospective cohort	4	2	0	2
Mongelli et al^28^	Cross-sectional cohort	4	2	0	2
Massa et al^29^	Cross-sectional cohort	4	2	0	2
Taylor et al^30^	Cross-sectional cohort	5	3	0	2
Cohen et al^31^	Retrospective cohort	5	2	0	3
Verdonck-de Leeuw et al^32^	Cross-sectional cohort	4	3	0	1
Short et al^33^	Retrospective cohort	5	3	0	2
Smith et al^34^	Retrospective cohort	4	2	0	2
Nilsen et al^35^	Cross-sectional cohort	4	2	0	2
Chaukar et al^36^	Cross-sectional cohort	5	3	0	2
Elting et al^37^	Prospective cohort	4	2	0	2
Mady et al^38^	Cross-sectional cohort	4	2	0	2
de Souza et al^39^	Cross-sectional cohort	3	1	0	2
Hueniken et al^40^	Cross-sectional cohort	5	2	0	3

Abbreviation: NOS, Newcastle-Ottawa Quality Assessment Scale.

## Results

The database searches and manual cross-reference search identified a total of 675 articles. After applying the PRISMA protocol criteria, a total of 33 studies were included in the literature review. There was heterogeneity in the reporting of financial burden for patients with HNC. Studies varied on reporting total healthcare costs over varying time frames, out-of-pocket costs, annual costs, median versus mean costs, or costs based on insurance status, etc, and thus, a qualitative synthesis of the studies was used to integrate study results by analyzing common themes present in the available literature. It was not possible to perform a meta-analysis on study results.

### Direct costs associated with HNC treatment

Seventeen studies included in the systematic review reported on direct costs associated with HNC treatment (Table 2).^2,6,9,13-26^ The median annual medical expenses were higher for patients with HNC than for patients with other cancers or controls.^2,6,9,13^ Lairson et al^13^ reported that patients with HNC paid US$139,749 more in total healthcare costs than those without cancer (*P* < .001): increased costs came from outpatient services (US$106,604), inpatient services (US$24,341), and prescription drugs (US$3,550). Major surgical procedures associated with HNC treatment increased the total health care costs,^14-16^ including costs for neck dissection and free-flap reconstruction.^14,17,18^

**Table 2.** Synthesis of direct costs experienced by HNC patients in published studies including those related to healthcare spending, inpatient and outpatient services, surgical procedures, hospital costs, out-of-pocket costs, treatment modality, and treatment-related complications.

Author	Study population	Comparisons/outcomes	Total spending	Spending differences
Jacobson et al^2^	6,812 cancer patients with OC/OP/SG cancer data in an administrative claims database compared with similar patients without cancer; retrospective cohort	Direct medical expenditures and indirect (short-term disability) expenditures during the year following the index date; charges for inpatient admissions, outpatient hospital visits, office visits, emergency department visits, outpatient prescription drugs	Total annual health care spending during the year after the index diagnosis: Commercial insurance, US$71 732 (*n* = 3,918); Medicare, US$35 890 (*n* = 2,303); Medicaid, US$44 541 (*n* = 585)	Single modality versus multimodal treatment (surgery, RT, and chemotherapy) Commercial: US$49,745 vs US$133,603 Medicare: US$35,932 vs US$77,860 Medicaid: US$54,804 vs US$99,666
Massa et al^6^	16,771 cancer patients and 489 with HNC; data extracted from the Medical Expenditure Panel Survey from 1998 to 2015; retrospective cohort	Demographics, income, employment, and health status; risk factors for medical expenses were assessed with regression modeling	Median annual medical expenses (US$8,384 vs US$5,978; difference, US$2,406; 95% CI: US$795-US$4,017]) and relative out-of-pocket expenses (3.93% vs 3.07%; difference, 0.86% [95% CI: 0.06-1.66]) were higher for patients with HNC than for patients with other cancers	Median expenses were lower for Asian individuals than for white individuals (US$5,359 vs US$10,078; difference, US$4,719 [95% CI: US$1,481-US$7,956]); lower for Westerners (US$8,094) and Midwesterners (US$5,656) than for Northwesterners (US$10,549); lower for those with excellent health than those with poor health (US$6,714 vs US$16,990)
de Souza et al^9^	73 treatment-naive patients with stage III, IVa, or IVb locally advanced HNC at a single institution tertiary care hospital from May 2013 to November 2014; prospective cohort	Self-reported data on demographics, income, wealth, cost-coping strategies, out-of-pocket costs, medication adherence, and social isolation	Median monthly direct costs were US$521 (range: US$0 to US$8,604).	Median monthly out-of-pocket cost was US$805.93 (range: US$6-US$10,156); median insurance deductible was US$750 (range: US$0-US$4,501). Median monthly insurance premium was US$211 (range: US$0-US$1,121).
Lairson et al^13^	467 patients diagnosed with OP cancer in 2011–2014 with commercial insurance claims in Texas and a control group of 467 noncancer patients obtained (propensity score matched); retrospective cohort	Total health care cost during the first 2 years after the index date	Adjusted mean total health care costs: cases, US$160,639; controls, US$20,890; Adjusted mean monthly cost: cases, US$6,693; controls, US$870	
Chan et al^14^	93,663 patients who underwent an ablative procedure for a malignant oral cavity, laryngeal, hypopharyngeal, or OP neoplasm in 2003–2008; retrospective cohort	Discharge data from the NIS database were analyzed using cross-tabulations and multivariate regression modeling	Increased hospital costs were significantly associated with urgent or emergent admission; Medicare, Medicaid, or self-pay status; comorbidity; neck dissection; major surgical procedures (mean, by US$15,528); and pedicled or free-flap reconstruction	
Hennessey et al^15^	93,663 patients who underwent an ablative procedure for a malignant oral cavity, laryngeal, hypopharyngeal, or OP neoplasm in 2003–2008; retrospective cohort	Discharge data from the NIS database were analyzed using cross-tabulations and multivariate regression modeling	DVT/PE cost an additional US$10,313 above the mean cost; major surgical procedure increased cost by a mean of US$14,690	DVT/PE was associated with increased risk of in-hospital mortality (odds ratio [OR], 3.1), postoperative surgical complications (OR, 2.1), acute medical complications (OR, 1.9) (all *P* < .001) and with increased LOS and hospital-related costs
Adjei Boakye et al^16^	71,440 patients with HNC; weighted hospital admissions from the 2014 NIS; retrospective cohort	Multivariable linear regression models estimated factors associated with hospitalization costs; negative binomial regression models were used to identify factors associated with hospital LOS	Average cost of HNC-related hospitalizations, US$20,985 (standard deviation [SD], US$23,318); total national cost, US$1.5 billion; the highest costs for admissions related to oral cavity (US$21,309) followed by larynx (US$18,843); major surgical operations increased cost by US$18,828 (95% CI: US$17,988-US$19,668); additional costs for chemotherapy (US$3,340 [95% CI: US$2,713-US$3,700]) and RT (US$7,406 [95% CI: US$5,935-US$8,878])	Admissions related to larynx (7.6 days) had the longest LOS followed by oral cavity (6.8 days); LOS and costs were associated with admissions involving bacterial infection, elective, major operating, chemo, and radiation procedures, at medium- or small-bed-number hospitals and rural hospitals, and among Black patients and those with advanced comorbidities
Jones et al^17^	100 consecutive patients undergoing microsurgical reconstruction for HNC at a single institution over a 5-year period; retrospective cohort	Actual costs of medical services were obtained by accessing the itemized “resource cost to the hospital” data	Average total cost of hospitalization, excluding professional fees, was US$35,099	Cost of occupying a hospital bed was directly related to LOS; ICU admission increased hospitalization fees from US$1,956 to US$9,760
Tom et al^18^	150 patients with various stage 0 to IVb HNC at a single tertiary care center in 2013; treatments consisted of surgery (17%), RT (11%), surgery + RT (14%), chemoradiation (45%), or surgery + chemoradiation (13%); retrospective cohort	Reported relative median costs scaled either as a ratio to a reference value or as a ratio relative to the MOC of treatment; multivariable linear regression to assess cost when both group stage and treatment type were considered	Treatment of stages II-IVa was on average 33% of the MOC more expensive than for stages 0-I (95% CI: 1.02 to 1.63; *P* = .04), and stage IVb was 60% of the MOC more expensive than stages 0-I (95% CI: 1.22 to 1.97; *P* < .001)	Increased costs were associated with higher stage, surgical reconstruction using a flap, use of a neck dissection, RT, chemotherapy, or weekly cisplatin, toxicity grade >3, unplanned hospitalization, use of a feeding tube, or any failure, distant metastasis, or death within 1 year; trimodal therapy (surgery + chemoradiation) had the highest cost, followed by chemoradiation, surgery + RT, RT alone, and surgery alone
Lang et al^19^	201 patients with advanced-stage SCC HNC assigned to either RT or RT + platinum-based chemoradiotherapy were followed up for 6 months; data from the PharMetrics Patient-Centric Database from June 2000 to June 2006; retrospective cohort	Measured clinical practices, incidence rates, and costs associated with treatment-related complications; assessed differences in total cost per patient and cost per treatment-related complication	Non-complication-related costs for therapy with and without chemoradiation: US$75,739 and US$50,677, respectively	
Arshad et al^20^	257 patients undergoing free-flap surgery for HNC at a single institution between 2006 and 2010; retrospective cohort	Chart review comparing flap outcomes, morbidity, and cost in patients with HNC-free flaps who recovered in the ICU versus those in a “specialty floor” setting	Cost per patient included laboratory (US$835), pharmacy (US$651), respiratory care (US$656), radiology (US$249), and supplies (US$224); ICU nursing (US$4,881)	Patients in the ICU group had additional costs (US$3,328) and longer LOS (mean, 10.28 vs 9.89; *P* = .008); postoperative complication increased the median LOS (from 8 to 10 days; *P* < .00001); no difference in the flap failure rates between the two groups
Moore et al^21^	76 patients with OP SCC treated at two hospitals from 2009 to 2010; retrospective cohort	Cost analysis of patient charts; TOS with neck dissection vs adjuvant radiation vs adjuvant chemoradiation vs primary CRT	Mean costs of private/government payers for TOS: US$37 435/US$15,664 (range: US$22,486-US$48,746/US$13,325-US$16,885 TOS + RT: US$74,484/US$34,343 (range: US$72,400-US$84,825/US$31,565-US$40 810); TOS + CRT: US$191,780/US$53,245 (range: US$145,450-US$217,220/US$49,400-US$58,325); CRT: US$198,285/US$57,429 (range: US$168,216-US$298,945/US$52,900-US$59,545)	
Brandenburg et al^22^	74 patients who underwent treatment for early glottic cancer (T1N0); retrospective cohort	Chart comparison of 44 patients treated with RT and 30 patients with endoscopic laser surgery	Endoscopic laser microresection of the tumor cost US$1,893, whereas RT cost US$29,353	
Nonzee et al^23^	99 patients with HNC (stages II-IV) and 40 patients with non-small lung cancer who had received radiochemotherapy; retrospective cohort	Chart review for presence/absence of severe mucositis/pharyngitis and the medical resources used; resource estimates were converted into cost units obtained from standardized sources (hospital bills, Medicare fee schedule, Red Book)	Total median medical costs per patient with and without mucositis/pharyngitis were US$39,313 and US$20,798, respectively (*P* = .007); incremental inpatient hospitalization costs for patients who had HNC with mucositis/pharyngitis were US$14,000, and total medical costs were US$17 244	
Kochhar et al^24^	123,662 patients who underwent an ablative procedure for a malignant oral cavity, laryngeal, hypopharyngeal, or OP neoplasm during 2001–2008; retrospective cohort	Discharge data from the NIS were analyzed using cross-tabulations and multivariate regression modeling	HAC were significantly associated with urgent or emergent admission (OR = 2.0, *P* = .004), major surgical procedures (OR = 2.3, *P* < .001), flap reconstruction (OR = 3.5, *P* < .001), and advanced comorbidity (OR = 2.0, *P* < .001). Hospital-acquired conditions were significantly associated with in-hospital mortality (OR = 3.8, *P* = .001) and surgical complications (OR = 4.9, *P* < .001).	
Pike et al^25^	454 patients with breast, ovarian, head/neck, or non-small cell lung cancer resulting in CAPN within 9 months of chemotherapy (cases) and those without CAPN (controls); retrospective cohort	Data obtained from an administrative claims database	Outpatient costs contributed the highest excess costs of US$8,092	Chemotherapy-associated peripheral neuropathy cost an extra US$36,660 per patient (primarily for outpatient costs); CAPN cases had 12 more outpatient visits than controls and spent more days in the hospital; work loss burden was higher for cases but not statistically different from controls.
Semenov et al^26^	93,663 patients who underwent an ablative procedure for a malignant oral cavity, laryngeal, hypopharyngeal, or OP neoplasm in 2003–2008; retrospective cohort	Discharge data from the NIS database were analyzed using cross-tabulations and multivariate regression modeling	Comorbidity, urgent or emergent admission, major surgical procedures, pedicled or free-flap reconstruction, Medicaid or self-pay payor status, dysphagia, and weight loss were significantly associated with increased hospital costs. Pneumonia from any cause was associated with significantly increased length of hospitalization and hospital-related costs, with infectious pneumonia having the single largest impact on both length of hospitalization and costs of care.	

Abbreviations: CAPN, chemotherapy-associated peripheral neuropathy; CI, confidence interval; CRT, chemoradiation therapy; DVT/PE, deep vein thrombosis/pulmonary embolism; HAC, hospital-acquired conditions; HNC, head and neck cancer; ICU, intensive care unit; LOS, length of stay; MOS, median overall cost; NIS, nationwide inpatient sample; OC, oral cavity; OP, oral pharyngeal; OR, odds ratio; RT, radiation therapy; SCC, squamous cell carcinoma; SD, standard deviation; SG, salivary gland; TOS, transoral surgery; UTI, urinary tract infection.

Five studies reported longer inpatient hospitalizations are a major contributor to the high costs of HNC treatment.^16,17,19-21^ Jones et al^17^ reported that the cost of a hospital bed accounted for 36.4% of total cost related to HNC treatment, while 24.5% of the total cost was for operating room fees. Postoperative ICU admission was an additional source of added cost.^17,20^

Four studies reported on the cost of treatment related to treatment modality.^2,18,19,21^ Surgery alone had the lowest cost, followed by radiation alone, surgery plus radiation, and chemoradiation, whereas trimodal therapy (including surgery and chemoradiation) was the most expensive.^2,18,19,21^ Costs associated with multimodality treatment were as high as US$153,892 in one study (twice the cost of single-mode therapy).^2^ Chemotherapy and radiation therapy increased the respective median overall costs 42% to 43% (*P* < .001) in Tom et al^18^ and five-fold and two-fold in Moore et al.^21^ Radiotherapy for early glottic cancer was 15.5 times more costly than endoscopic laser surgery in Brandenburg et al.^22^ Lang et al^19^ reported that radiotherapy alone (US$56,900) was less costly than multimodal therapy with chemoradiotherapy (US$91,564) and had lower 6-month complication-related health care costs (US$6,223) than chemoradiotherapy (US$15,825).

Ten studies reported on complications related to HNC treatment and the resultant increase in health care costs, often the results of prolonged hospitalization stays.^14-17,19,20,23-26^ Chemotherapy-related complications were specifically associated with significant increases in health care costs in three studies.^16,19,25^ For example, Lang et al^19^ reported that chemotherapy complications cost an extra US$14,714, primarily for inpatient costs: treatment-related complications occurred in 86% of patients treated with chemoradiotherapy versus 51% in those who were treated with radiotherapy (*P* < .001); oral complications, dehydration, and electrolyte imbalances were the most expensive complications and were more common (*P* < .001) among patients who received chemoradiotherapy. Nonzee et al^23^ reported 70% (70/99) of patients with HNC treated with chemoradiotherapy had severe mucositis as a treatment-related complication, which resulted in increased medical costs (US$12,600) due to extended inpatient hospitalization (median 14 days [US$19,600] vs 5 days [US$7,000] for those without this complication; *P* = .017). Jones et al^17^ reported that patients with a postsurgical complication had a 70.7% increase (from US$28,706 to US$48,998) in total resource cost and a prolonged hospital stay (24 days vs 13.5 days) compared with those without complications. Kochhar et al^24^ found hospital costs increased by US$20,211 for complications from hospital-acquired conditions. Other complications included urinary tract infection (US$14,992),^14^ deep venous thrombosis (US$10,313),^15^ central line- and vascular catheter-associated blood infections (US$14,616 and US$22,757, respectively),^24^ infectious pneumonia (US$17,095), aspiration pneumonia (US$12,510), weight loss (US$7,739), dysphagia (US$2,609),^26^ retained foreign body after surgery (US$18,968), and fall/trauma (US$5,655).^24^

### Indirect costs associated with HNC treatment

Eleven studies included in the systematic review reported on indirect costs associated with HNC treatment (Table 3).^2,6,9,28-34,40^ Eight studies reported on issues surrounding employment and disability induced by their cancer treatment.^2,27-33^ Short et al^33^ found that the risk of disability or quitting work was higher in patients with HNC than in those with other cancers (odds ratio [OR], 1.713; 95% CI: 0.684-4.293), particularly for female patients (OR, 3.392; 95% CI: 1.018-11.299). Taylor et al^30^ found that 52% (201/384) of patients were disabled by their cancer treatment, with greater risk from chemotherapy (OR, 3.4) or a neck dissection (OR, 2.3) (*P* < .05).^30^ Beeler et al^27^ reported that 69% of patients stopped working during radiation therapy, whereas Massa et al^29^ reported that 26% of patients stopped working after undergoing multimodality treatment. Verdonck-de Leeuw et al^32^ found that 17% of patients had not returned to work 2 years after curative treatment for HNC. Among patients with thyroid cancer, the unemployment rate was 12.3% (corresponding national unemployment rate, 4.4%).^28^

**Table 3.** Synthesis of indirect costs experienced by HNC patients in published studies including effects of unemployment, disability, and decreased work productivity.

Author	Study population	Comparisons/outcomes	Objective cost
Jacobson et al^2^	6,812 patients with OC/OP/SG cancer; data from an administrative claims database; retrospective cohort	Direct medical expenditures and indirect (STD) expenditures during the year after the index date; charges for inpatient admissions, outpatient hospital, office, and emergency department visits, and outpatient prescription drugs	US$17,876 in STD costs vs US$6,916 for a matched comparison group (*P* < .01) 48.3 days was the average number of disability days during the first year after the diagnosis.
Massa et al^6^	16,771 patients with cancer (489 with HNC); data from the Medical Expenditure Panel Survey from 1998 to 2015; retrospective cohort	Demographics, income, employment, and health were compared between patients with HNC and those with other cancers; risk factors for medical expenses were assessed with regression modeling	Higher relative out-of-pocket expenses were associated with unemployment (5.13% vs 2.35% for employed patients; difference, 2.78% [95% CI: 0.6-4.95]), public insurance (5.35% vs 2.87% for those with private insurance; difference, 2.48% [95% CI: −0.6 to 5.55]), poverty (13.07% vs 2.06% for high-income patients), and poor health status (10.2% vs 1.58% for those with good health). Patients with HNC were more often than those with other cancers to be members of a minority race/ethnicity, male, poor, publicly insured, less educated, with lower general and mental health status
de Souza et al^9^	73 patients with treatment-naive stage III, IVa, or IVb locally advanced HNC at a single institution tertiary care hospital from May 2013 to November 2014; prospective cohort	Self-reported data on demographics, income, wealth, cost-coping strategies, out-of-pocket costs, medication adherence, and social isolation	Median monthly direct nonmedical cost was US$42 (range: US$0-US$948); median financial burden (out-of-pocket/household income) was 15.1% (range: 0.5%-192,400%)
Mongelli et al^28^	1,743 survivors of thyroid cancer; cross-sectional cohort	Online financial distress questionnaire and patient-reported outcomes measurement system (29-item)	18.1% (315) reported unemployment for 6 months or more due to thyroid cancer or related treatment. 59.6% (1,033) lost productivity at work, 42.5% (737) lost income at work, and 73.7% (1,279) took time away from work due to their thyroid cancer diagnosis or related treatment.
Massa et al^29^	100 patients with HNC at a tertiary clinic: 67 had advanced-stage disease and 70 received multimodal therapy; cross-sectional cohort	15-question survey evaluating the logistic, financial, and psychosocial burdens associated with clinic visits administered a median of 18 months after treatment completion	51% were employed prior to their diagnosis, which decreased to 25% following treatment. Of the patients that continued to work after treatment, most (51.8%) missed 1 day of work to attend clinic visits; 19% of patients relied on public or medical transportation; treatments and tests were most often the greatest cause of financial burden (35% and 29%, respectively), whereas tests and transportation were the greatest cause of stress (31% and 27%, respectively). Largest proportion of stress was attributable to tumor recurrence (59.7%), followed by cost of medical expenses (38.9%) and travel (30.6%)
Taylor et al^30^	384 patients with HNC in a multisite study using survey and chart data to determine the predictors of work-related disability; cross-sectional cohort	HNQOL questionnaire, pain domain was included as the only QOL variable; Fagerstrom test for nicotine dependence, AUDIT, and GDS-SF	Patients who smoked, were depressed, and/or had less education had greater disability than those without these characteristics (*P* < .05). Multivariate analysis linked disability with pain scores (OR, 1.2; *P* = .01) and time since diagnosis (OR, 0.9; *P* = .04)
Cohen et al^31^	386 patients with 12 months of follow-up who had an STD claim linked to a dysphonia diagnosis (ICD-9 code) between January 1,2004 and December 31, 2008; retrospective cohort	National database of work absence	Mean of 39.2 (95% CI: 31.9-46.5) days absent from work; total STD payments in 2008 dollars were US$647,269, with a mean per person in 12 months of US$3,407. Total and mean lost wages in 12 months were US$843,199 and US$4,438, respectively
Verdonck-de Leeuw et al^32^	85 patients younger than 65 years at time of diagnosis and at least 2 years after curative treatment for HNC; cross-sectional cohort	Primary outcome measures were employment status and return to work assessed by questionnaire. Secondary outcome measures were HRQOL (EORTC QLQ-C30 and QLQ-H&N35) and emotional distress (HADS)	Of the 44 patients who returned to work after treatment, 28 returned to the same work, 7 to adapted work, and 9 to other work; patients with advanced-staged HNC more often changed work or did not return to work Median time to return to work was 6 months (range: 0-24 months), and 71% of patients returned to work within 6 months after treatment; Significant barriers to employment after treatment were anxiety, oral dysfunction, xerostomia, trismus, sticky saliva, problems with teeth, loss of appetite, problems with social eating, and social contacts
Short et al^33^	1,433 cancer survivors (58 with HNC) who were working when they were diagnosed from 1997 to 1999; retrospective cohort	Phone interviews regarding employment and work-related disability from 1 to 5 years after diagnosis	41% males and 39% females who were working at the time of diagnosis stopped during cancer treatment. 21% of the females and 16% of the males who were working at diagnosis, reported limitations in ability to work that were related to cancer. Of the survivors who returned to work in the first year, 11% would quit for cancer-related reasons in the next 3 years. New cancers or metastases increased the likelihood of quitting work (OR, 2.101; *P* = .0012). More advanced disease was associated with greater employment challenges for survivors.
Smith et al^34^	101 patients with carcinoma *in situ* and T1 invasive SCC treated with surgery or RT; retrospective cohort	Two validated questionnaires (modified UWQOL and PSS-HN) and one local questionnaire (hidden cost questions related to treatment, such as travel time, distance, treatment time, and work missed)	Patient-reported problems (swallowing, chewing, speech, taste, saliva, pain, activity, recreation, and appearance) did not differ among those with surgery or RT
Hueniken et al^40^	430 patients with HNC at 12 to 24 months after treatment; cross-sectional cohort	Validity was tested using the Spearman ρ^a^; responsiveness analysis compared change in income and change in FIT between 12 and 24 months	Change in income was negatively correlated with change in FIT over time (ρ = −0.25; *P* = .04).

Abbreviations: AUDIT, alcohol use disorders identification test; CI, confidence interval; OC, oral cavity; OP, oral pharyngeal; EORTC, European organization for research and treatment of cancer; FIT, financial index of toxicity; GDS-SF, geriatric depression scale–short form; HADS, hospital anxiety and depression scale; HNC, head and neck cancer; HNQOL, head and neck quality of life; HRQOL, health-related quality of life; ICD, International Classification of Diseases; OR, odds ratio; PSS-HN, performance status scale for head and neck cancer; QLQ-C30, Quality of Life Questionnaire subscale of the EORTC with cancer-specific questionnaire for measuring HRQOL and 5 functional scales; QLQ-H&N35, Quality of Life Questionnaire-Head and Neck Module subscale of the EORTC that measures HNC specific issues with 7 subscales; QOL, quality of life; RT, radiation therapy; SCC, squamous cell carcinoma; SDI, social difficulties inventory; SG, salivary gland; STD, short-term disability; UWQOL, University of Washington Quality of Life.

a See Table 5 for details.

For many patients that were able to continue working, there was lost productivity from missed work for clinic visits, reduced work hours, and use of unpaid time off and sick leave.^27,29^ Massa et al,^29^ reported that patients’ greatest financial and stress burdens (13% and 18%, respectively) were from lost income as a result of absence from work. Mongelli et al^28^ found that lost productivity at work was significantly (*P* < .01) associated with worse fatigue (β = 5.99) and social functioning (β = 4.07). Issues with social eating and anxiety from oral dysfunction in some HNC survivors were identified as barriers to employment.^19,23,32^ In one study, patients with oral, oropharyngeal, and salivary gland cancers who continued to work used 44.9 more short-term disability days than other employees (*P* < .01).^2^ In a study by Cohen et al,^31^ patients with laryngeal cancer had the highest short-term disability payments (average, US$9,544 [SD, US$8,005]) and had the most days absent from work (average, 98 days [SD, 65 days]). Smith et al^34^ studied patients with early glottic cancer and found that those treated with radiation therapy required significantly (*P* < .01) more treatments (35 vs 2) and had more hours of missed work (76 vs 24), more travel time (1,440 vs 180 min), and greater travel distances (660 vs 150 miles) than patients treated with endoscopic excision. Impressively, in the study of 73 patients with locally advanced HNC reported by de Souza et al,^9^ lifetime lost wages were as much as US$1,317,882, with a median of US$135,271.

### Consequences of FT

Ten studies included in the systematic review reported on consequences of FT associated with HNC treatment (Table 4).^3,8,9,27,28,35-39^ Four studies reported that the HRQOL for HNC patients significantly declined because of their health status and treatment-related social function, appetite, sleep, pain, mucositis, and dysphagia.^8,35-37^ Four studies reported on depression, anxiety, or stress related to cancer treatment.^3,28,29,35^ Chen et al^3^ found that 17% (36/211) of patients treated with radiation for SCC HNC had depression 1 year later (related to smoking, feeding tube dependence, and presence of tracheostomy or laryngeal stoma). Mongelli et al^28^ reported that financial difficulties for 43% of thyroid cancer survivors were associated with worse anxiety (β = 5.07; *P* < .01) and depression (β = 5.47; *P* < .01). Nilsen et al^35^ reported that of 228 survivors of HNC, 132 (56%) reported that treatment-related symptoms of depression and anxiety impacted their daily life. In the study by Massa et al,^29^ 38.9% of the stress reported by patients with HNC was attributable to medical expenses, while transportation concerns were the greatest source of stress for 27 patients and surgery with adjuvant chemoradiation was reported as more stressful than surgery alone.

**Table 4.** Synthesis of consequences of FT including impact on QOL and use of cost-coping strategies.

Author	Study population	Comparisons/outcomes	Subjective cost
Chen et al^3^	211 patients with SCC HNC treated with RT that were disease-free with at least 1 year of follow-up; cross-sectional cohort	UWQOL instrument survey	Patients who reported their mood as “somewhat depressed” and “extremely depressed” at 1 year after completion of RT was 12% and 5%, respectively; at 3 years, 8% and 7%; at 5 years, 9% and 4%. The proportion of patients using antidepressant medications was 6% (2 of 36), 11% (2 of 18), and 0% (0 of 7), at 1, 3, and 5 years.
Tribius et al^8^	161 patients with locally advanced HNC at the end of RT and at 12- and 24-month follow-up; prospective cohort	Questionnaires of the EORTC (QLQ-30 and QLQ-HN35) 2 years after RT	Middle- and low-SES patients reported lower QOL for global health status, physical function, and role function and higher general (fatigue, pain, dyspnea) and HNC-specific (pain, swallowing, senses, speech, social eating, opening mouth, and felt ill) symptom burdens than patients with high SES Patients with high-SES status report worse QOL index at the end of RT in the domains of global health status (–15.2), role function (–23.8), and social function (–19.4) than patients with middle and low SES (all *P* < .05); QOL improved during the first 12 and 24 months.
de Souza et al^9^	73 patients with treatment-naive stage III, IVa, or IVb locally advanced HNC at a single institution tertiary care hospital from May 2013 to November 2014; prospective cohort	Self-reported data on demographics, income, wealth, cost-coping strategies, out-of-pocket costs, medication adherence, and social isolation	Medicaid patients were more likely to use cost-coping-strategies, associated with decreased wealth and higher total out-of-pocket costs (*P* < .01). Patients with high perceived social isolation were more likely to use cost-coping strategies (OR, 11.5), to report nonadherance to supportive medications (21.4 vs 5.45 days over 6 months), and to miss appointments (7 vs 3) (*P* ⩽ .01)
Beeler et al^27^	63 patients with HNC within 12 months from completion of definitive, postoperative, or palliative RT; prospective cohort	Survey using the COST tool	Median COST score was 26.5 (0-44), indicating moderate impact of FT on QOL; younger age, lower income, and unemployment/disability were associated with greater FT (*P* < .05). Patients who continued working used sick leave, took unpaid time off, or arranged to work fewer hours; patients with lower COST scores were more likely to report worry and financial burden (*P* < .0001) and to use savings or borrow money, skip clinic visits, or not fill prescriptions because of the cost
Mongelli et al^28^	1,743 survivors of thyroid cancer; cross-sectional cohort	Online financial distress questionnaire and patient-reported outcomes measurement system (29-item)	23.7% (413) used up all or most of their savings; 15.1% (263) borrowed money from friends or relatives; 3% (53) declared bankruptcy; 12% (209) reached their maximum credit card limit; 4.4% (76) had to take out a new loan or mortgage; and 15.9% (277) had been contacted by a collection agency. Those who reported financial distress also reported worse anxiety and depression (*P* < .0001). Respondents who reported they were unable to change jobs or get a new job due to their thyroid cancer diagnosis reported worse fatigue, pain interference, and social functioning (*P* < .0001). Those who endorsed higher financial difficulty and financial distress were predicted to have worse HRQOL in all 7 PROMIS domains (*P* < .0001).
Nilsen et al^35^	228 survivors attending a multidisciplinary HNC survivorship clinic; cross-sectional cohort	QOL was measured using the 16-item UWQOL version 4. Primary outcomes were QOL, symptoms of anxiety and depression (assessed with PHQ-8 and GAD-7), and swallowing dysfunction (examined with the EAT-10)	Advanced-stage disease at diagnosis (stages III-IV) was associated with severe swallowing dysfunction (*P* = .004) 41 (18.1%) survivors were at risk for depression; 26 (11.5%) had symptoms of major depression; 29 (12.9%) were at risk for anxiety; and 22 (9.8%) had symptoms of generalized anxiety disorder; male gender, longer time since treatment, and treatment with surgery alone were associated with higher physical QOL (*P* < .05) and social-emotional QOL (*P* < .05)
Chaukar et al^36^	212 HNC survivors 1 year after completion of treatment at a tertiary cancer center; cross-sectional cohort	QOL assessments using EORTC QOL questionnaire core-30 and the HNC module.	HNC survivors reported a high incidence of financial difficulties (54%), appetite loss (36%), fatigue (33%), and cough (30%); QOL was impacted negatively by dry mouth (64%), dental problems (42%), sticky saliva (40%), cough (39%), and problems with mouth opening (32%); patients with early-stage tumors or treated with surgery alone had better QOL scores than those with advanced-stage tumors or receiving RT alone or multimodality treatment, respectively
Elting et al^37^	206 patients with SCC of the oral cavity, OP, larynx, or hypopharynx (stages I-III) who received a cumulative dose of least 40 Gy of RT; prospective cohort	Validated, patient-reported questionnaire (MDQ), the FACT QOL, and the FACIT fatigue scales to measure mucositis (reported as mouth and throat soreness), daily functioning, and use of analgesics; patients were studied before, daily during, and for 4 weeks after RT	Mean QOL score decreased from 85.1 to 69.0 at week 6 of RT, corresponding with the peak of mucositis severity; the mean functional status score decreased 33% (from 18.3 at baseline to 12.3 at week 6) Impact of mucositis on QOL was proportional to its severity; mild or moderate severity was associated with reduced QOL (from 93.6 at baseline to 74.7 at week 6)
Mady et al^38^	104 insured patients at a tertiary multidisciplinary HNC survivorship clinic who completed treatment for SCC of the OC, OP, or larynx/hypopharynx from Jan to April 2018; cross-sectional cohort	COST and FDQ; HRQOL was assessed by the UWQOL version 4	30 survivors demonstrated high FT; patients with worse FT were more likely not married (COST, 25.33 vs 30.61), of lower education levels (COST, 26.12 vs 34.14), and with primary cancers of the larynx/hypopharynx (COST, 22.86 vs 30.27 vs 32.72) (all *P* < .01) COST was associated with HRQOL (0.08, *P* = .03); 63 of 102 survivors reported using savings and/or loans; worse FT was associated with using more cost-coping mechanisms, younger age (OR, 4.23), lower earnings at diagnosis (OR, 1.17), and loss in earnings (OR, –1.80) (all *P* < .01)
de Souza et al^39^	233 patients with stage IV solid tumors receiving chemotherapy for ⩽2 months; cross-sectional cohort	COST measure; patient characteristics, clinical trial participation, health care use, willingness to discuss costs, psychological distress (POMS), and HRQOL (measured by the FACT-G and EORTC QOL questionnaires).	COST values correlated with income psychosocial distress^a^ Independent factors associated with FT were race, employment status, income, no. of inpatient admissions, and psychological distress (all *P* < .01)

Abbreviations: COST, comprehensive score for financial toxicity; EAT-10, eating assessment tool-10; EORTC, European Organization for Research and Treatment of Cancer; FACIT, functional assessment of chronic illness therapy; FACT-G, functional assessment of cancer therapy-general; FDQ, financial distress questionnaire; FT, financial toxicity; GAD-7, generalized anxiety disorder questionaire-7; HNC, head and neck cancer; HRQOL, health-related quality of life; MDQ, Mood Disorder Questionnaire; OC, oral cavity; OP, oral pharyngeal; PHQ-8, patient health questionaire-8; POMS, brief profile of mood states; PROMIS, Patient-Reported Outcomes Measurement Information System; QLQ-HN, Quality of Life Questionnaire-Head and Neck Module; QOL, quality of life; RT, radiation therapy; SCC, squamous cell carcinoma; SES, socioeconomic status; SG, salivary gland; UWQOL, University of Washington Quality of Life.

a See Table 5 for details.

Four studies reported on “cost-coping strategies” used by patients with HNC, including taking out loans, borrowing money, delaying or not filling prescription medications, and refusing recommended interventions.^9,27,38,39^ de Souza et al^9^ noted that 51 of 73 (69%) patients with advanced HNC used at least one cost-coping strategy.

### Assessment of FT in HNC

Several instruments have been developed to assess a patient’s financial burden in relation to their cancer treatment; however, we only identified two instruments that were used to assess financial burden specifically in patients with HNC. Table 5 summarizes comparative metrics for these two tools: the COST and the FIT.

**Table 5.** Comparison of COST and FIT surveys.

Metric	COST survey		FIT survey
Validation (no. of patients)	155^a^ and 233^b^		430^c^
Internal consistency (Cronbach’s α)	0.92		0.77
Test-retest reliability (intraclass correlation) [95% CI]	0.80 [0.57-0.92]		0.70 [0.38-0.87]
Construct and concurrent or convergent validity			
Correlated with:	Higher psychological distress	Worse HRQOL^c^	Financial difficulties
Index	POMS (*r* = 0.26, *P* < .001)	FACT-G (*r* = 0.42, *P* < .001); EORTC QOL questionnaire (*r* = 0.33, *P* < .001)	SDI, money matters subscale (ρ = 0.374, *P* = .008)^d^
Inverse relationship between scores and income	FT vs household income (*r* = 0.28, *P* < .001)		FIT scores vs income (ρ = −0.337, *P* < .001); FIT scores vs lost income (ρ = 0.243, *P* < .001)

Abbreviations: CI, confidence interval; COST, comprehensive score for financial toxicity; EORTC, European organization for research and treatment of cancer; FACT-G, functional assessment of cancer therapy-general; FIT, financial index of toxicity; FT, financial toxicity; HRQOL, health-related quality of life; POMS, Brief profile of mood states; QOL, quality of life; SDI, social difficulties inventory.

a Patients with advanced (TNM stage IV) solid organ cancers who were treated with chemotherapy in the United States.^41^

b Patients with advanced (TNM stage IV) solid organ cancers who were treated with chemotherapy in the United States.^39^

c Patient HNC of multiple stages who were treated with various treatment modalities in Canada.^40^

d Tested in 49 patients.

The COST survey was developed and validated by de Souza et al^41^ in 2014 in a group of 155 patients and was studied again in 2017 in 233 patients;^39^ both groups had advanced-stage IV solid tumor cancers and were receiving chemotherapy. The authors developed an 11-item survey, with each item graded on a 5-point Likert scale (0 to 4): total scores range from 0 to 44, and lower scores indicate worse FT. The tool is meant to assess patient-reported financial distress and how this impacts their lives.

The COST survey has been used in two published studies in patients with HNC.^27,38^ In a study of 64 patients with HNC treated with radiation therapy by Beeler et al,^27^ patients with greater FT (indicated by lower COST scores) exhibited cost-coping strategies: 54% decreased spending on food and clothing, 43% used savings, and 13% borrowed money to pay for treatment. Patients with lower COST scores and greater FT were significantly more likely to refuse recommended tests, miss clinic visits, and not take medications as prescribed.^27^ Patients who required a feeding tube and supportive infusions were statistically more likely to have a lower COST score and greater FT, suggesting FT is associated with a higher risk for morbidity.^27^ Patients with lower COST scores were also significantly more likely to report worry and financial burden.^27^ Mady et al^38^ found that 30 of 104 (28.8%) survivors of HNC had high FT and that COST was associated with HRQOL; 60% of the survivors reported using cost-coping mechanisms. In these two studies, lower COST scores and worse FT were associated with younger age, lower income, single individuals, lower education levels, and the larynx as the primary site of cancer.^27,38^

The FIT was developed and validated by Hueniken et al^40^ in 2020 in 430 patients with HNC (of multiple stages and who were treated with multiple treatment modalities) 12 to 24 months after treatment at a single institution in Canada. The FIT incorporates a nine-item survey to measure three subdomains of FT: financial stress, financial strain, and lost productivity. Each item is graded on a scale from 0 to 1, and the total score is calculated from the mean scores of all responses and multiplied by 100; total scores range from 0 to 100, and higher scores represent worse FT. Their study demonstrated that worse FT and higher FIT scores correlated with a lower baseline household income and higher amount of lost wages.^40^ This initial study shows promise for the use of the FIT survey in the evaluation of patients with HNC; however, it has not been validated in further published studies.

## Discussion

The reviewed evidence indicates that medical expenses are higher for patients with HNC than for patients with other cancers.^2,6,13^ The added costs were attributable to major surgical procedures, multimodal treatment (trimodal therapy was the most expensive), treatment-related complications (particularly for chemotherapy and radiation therapy), and longer hospital stays.^2,14-19,21^ The indirect costs contributing to FT in patients with HNC reflect the long-term side effects of treatment, including lost employment/productivity as well as impacts on important daily and social functions such as swallowing, eating, speech, and communication. Additional burdens of depression, anxiety, and social isolation related to HNC treatment reduce treatment adherence and HRQOL.

Two tools were reported for the quantitative assessment of FT in patients with HNC: the FIT and the COST. The FIT survey was used in one Canadian study of patients with HNC, and the COST survey was validated in several studies in the United States in patients with advanced-stage IV solid tumor cancers and employed in two original research studies of HNC patients. The COST survey demonstrated internal consistency, test-retest reliability, and concurrent validity for 233 patients via three indices, whereas the FIT test demonstrated concurrent validity for 49 patients via one inventory.

The current models of care will be increasingly inadequate to meet the needs of the growing population of cancer survivors. Thus, novel strategies are necessary to better address these needs. Cancer centers, physicians, health insurance providers, and patients all play important roles in mitigating the effects of FT. Healthcare systems should be more transparent about the cost of treatment and provide better access to designated cancer centers. Physicians can engage in patient-centered cancer care and follow professional cancer society guidelines to avoid low-value interventions. Physicians can also make a point to discuss patient goals and lifestyle changes as well as designate a member of the health care team to help with early referrals to supportive care services. Recently, the use of telemedicine rather than in-person clinic appointments has shown utility in reducing costs as well.^42,43^ Insurance providers can restructure cost-sharing plans and offer more payment options and financial assistance. Patients should ideally be fully engaged in the formation of their treatment goals and be self-motivated in seeking further education and counseling.

Early interventions can lead to long-term reduction in health care costs and improve patient outcomes. A study by Yarlagadda et al^44^ aimed to better prepare patients for lifestyle changes during the treatment of HNC through preadmission social work counseling, which improved patient-reported feelings of support during hospitalization and treatment. Social work counseling that continued throughout treatment and discharge reduced patient anxiety and stress, improved perceived communication, and facilitated earlier access to resources.^44^ Patients who received this counseling were better prepared for instructions from nurses, more motivated, and had a more timely discharge.^44^

Patients that have better access to information regarding support groups, financial advice, and the long-term effects of treatment on the ability to work, physical functioning, and QOL have shown reduced feelings of isolation, anxiety, and depression, leading to better outcomes.^44-50^ A study evaluating the perspectives of patients with HNC on the cost of care revealed that patients preferred having a conversation, particularly with insurance representatives and hospital-based financial advisors, about the costs before initiating treatment.^49^

Multidisciplinary HNC clinics have been shown to help coordinate care by allowing patients to see multiple providers during the same visit, reducing the overall time off from work for follow-up care while also facilitating treatment adherence.^45,51^ Multidisciplinary cancer care also emphasizes rehabilitation interventions with the inclusion of physical and occupational therapy, speech therapy, and vocational rehabilitation, helping survivors return to work and thus ease economic burden. ^52-54^ Multidisciplinary cancer treatment teams may also include a specialized patient navigator or financial advisor who can help patients and caregivers cope with financial burdens.^45^

## Limitations

Although the literature search was comprehensive, this review is subject to low-quality evidence of available literature and publication bias. Interpretations of FT reflect patients’ subjective experience and may be dependent on the cancer type, treatment regimen, and variability in provider and patient preferences, which may lead to confounding bias and decreased homogeneity of results. Only two tools have been reported to quantify FT in cancer patients, which need further validation. Finally, because of the heterogeneity among studies, we were unable to conduct a meta-analysis of the data.

## Conclusions

Financial toxicity is an important consideration for the well-being of patients with HNC. More widespread use of screening and measurement tools, such as the COST and FIT, will raise awareness of the FT endured by these patients. Clinics should have referral resources and strategies available to address these issues to mitigate the economic burden of cancer care, which will improve the QOL for cancer survivors.

## Supplemental Material

sj-docx-1-onc-10.1177_11795549221147730 – Supplemental material for Understanding Financial Toxicity in Patients with Head and Neck Cancer: A Systematic Review Click here for additional data file. Supplemental material, sj-docx-1-onc-10.1177_11795549221147730 for Understanding Financial Toxicity in Patients with Head and Neck Cancer: A Systematic Review by Mattie Rosi-Schumacher, Shivam Patel, Chandat Phan and Neerav Goyal in Clinical Medicine Insights: Oncology
