# Supplementary material for: Understanding Financial Toxicity in Patients with Head and Neck Cancer: A Systematic Review
Source: Clin Med Insights Oncol. 2023 Jan 23;17:11795549221147730. doi: 10.1177/11795549221147730 (PMC9880590; doi:10.1177/11795549221147730)
Supplement: sj-docx-1-onc-10.1177_11795549221147730 – Supplemental material for Understanding Financial Toxicity in Patients with Head and Neck Cancer: A Systematic Review [file sj-docx-1-onc-10.1177_11795549221147730.docx]

Database search terms

1. *PUBMED*

("head and neck neoplasms"[MeSH Terms] OR "head and neck neoplasms"[MeSH Terms] OR "head and neck neoplasms"[MeSH Terms] OR "head and neck neoplasms"[MeSH Terms] OR "head and neck neoplasms"[MeSH Terms] OR ("head and neck cancer"[Title/Abstract] OR "head and neck cancers"[Title/Abstract] OR "head and neck neoplasm"[Title/Abstract] OR "head and neck neoplasms"[Title/Abstract] OR "head and neck carcinoma"[Title/Abstract] OR "head and neck carcinomas"[Title/Abstract])) AND ("burden of treatment"[Title/Abstract] OR "financial burden"[Title/Abstract] OR "financial toxicity"[Title/Abstract] OR "financial effects"[Title/Abstract] OR "financial impact"[Title/Abstract] OR "financial hardship"[Title/Abstract] OR "healthcare costs"[Title/Abstract] OR "healthcare cost"[Title/Abstract] OR "cost of treatment"[Title/Abstract] OR "cost of illness"[Title/Abstract] OR "health expenditure"[Title/Abstract] OR "economic burden"[Title/Abstract] OR "cost of illness"[MeSH Terms])

1. *EMBASE*

'head and neck cancer*':ti,ab OR 'head and neck tumor*':ti,ab OR 'head and neck surgery':ti,ab OR 'head and neck disease*':ti,ab OR 'head and neck squamous cell carcinoma*':ti,ab OR 'head and neck neoplasm*':ti,ab OR 'cancer of the head and neck':ti,ab OR 'cancer of head and neck':ti,ab OR 'head and neck carcinoma*':ti,ab

*AND*

'burden of treatment':ti,ab OR 'financial burden':ti,ab OR 'financial toxicity':ti,ab OR 'financial effects':ti,ab OR 'financial impact':ti,ab OR 'financial hardship':ti,ab OR 'healthcare costs':ti,ab OR 'healthcare cost':ti,ab OR 'cost of treatment':ti,ab OR 'health expenditure':ti,ab OR 'economic burden':ti,ab OR 'cost of illness':ti,ab

1. *Web of Science*

(TS= (head and neck cancers OR head and neck cancer OR head and neck neoplasm OR head and neck neoplasms) AND (TS= (Cost of illness OR financial toxicity OR financial burden OR financially viable OR financial viability OR ability to pay OR financial implications OR economic burden OR economic hardship OR financial distress OR financial hardship OR financial stress OR financial toxicity.

1. *Cochrane Library*

(Cost of illness OR “financial toxicity” OR “financial burden” OR “financially viable” OR “financial viability” OR “ability to pay” OR “financial implications” OR “economic burden” OR “economic hardship” OR “financial distress” OR “financial hardship” OR “financial stress” OR ‘”financial toxicity”) AND (head neoplasms OR head cancer OR neck neoplasm OR neck cancer)
